# Prognostic Value of Urine Sodium in Patients With Acute Decompensated Heart Failure: A Systematic Review and Meta-Analysis

**DOI:** 10.7759/cureus.110663

**Published:** 2026-06-11

**Authors:** Muftawu-Deen Iddrisu, Esteban Rodriguez, Xigmara Martinez Leyva, Olufunmilayo Obisesan, Ethan Molitch-Hou, Sonya Hui, Ann Nguyen

**Affiliations:** 1 Hospital Medicine, University of Chicago Medicine, Chicago, USA; 2 Internal Medicine, Ascension Saint Joseph, Chicago, USA; 3 Medicine, University of Medical Sciences of Camagüey, Camaguey, CUB; 4 Cardiovascular Medicine, University of Chicago Medicine, Chicago, USA

**Keywords:** acute exacerbation of heart failure, heart failure, heart failure readmissions, length of hospitalization, reduce mortality rate, spot urine

## Abstract

Acute decompensated heart failure (ADHF) remains a leading cause of hospitalization worldwide. The prognostic value of spot urinary sodium concentration in determining clinical outcomes for patients with ADHF treated with diuretics remains uncertain.

This systematic review with meta-analysis was registered in the International Prospective Register of Systematic Reviews (CRD42024626912). MEDLINE, Cochrane, and Embase were searched from inception to May 2026 for studies comparing high and low urine sodium, measured after the first void or within six hours of diuretic use, in adult patients admitted for ADHF. The primary outcomes of interest were mortality, length of hospitalization/stay (LOS), inotrope use, and worsening renal function. Odds ratios (ORs) with 95% confidence intervals (CI) were pooled with a random-effects model. Quality assessment and risk of bias were performed according to Cochrane recommendations.

A total of eight studies comprising 1,359 patients were included in the meta-analysis, of which 923 (67.9%) were categorized in the high urinary sodium group and 464 (32.1%) in the low urinary sodium group. In patients with ADHF, high urine sodium was associated with lower mortality (OR, 0.28; 95% CI, 0.12-0.68), reduced inotrope use (OR, 0.40; 95% CI, 0.25-0.62), lower worsening renal function (OR, 0.53; 95% CI, 0.32-0.86), and shorter LOS (mean difference, -4.35; 95% CI, -7.88 to -0.81) compared with low urine sodium.

In patients with ADHF treated with diuretics, high urine sodium is associated with improved outcomes, including reduced mortality, inotrope use, worsening renal function, and shorter LOS, compared with patients with low urine sodium.

## Introduction and background

Acute decompensated heart failure (ADHF) is defined by the sudden onset or worsening of heart failure symptoms, often requiring hospitalization and intensive management [1,2]. It represents a major global health burden, associated with high morbidity and mortality, frequent hospital admissions, and considerable healthcare costs [3]. Accurate prognostic evaluation and risk stratification are essential for informing clinical decision-making and improving patient outcomes [4-6]. Established tools, such as the Seattle Heart Failure Model score, New York Heart Association functional class, and biomarkers like natriuretic peptides, have been instrumental in assessing ADHF [4-6]. There is increasing interest in other accessible biomarkers that provide additional prognostic insight for patients with heart failure [6,7].

In ADHF, reduced renal blood flow and venous congestion trigger neurohormonal activation, leading to increased sodium and water reabsorption, primarily due to elevated aldosterone and angiotensin II levels. This decreases sodium delivery to the distal nephron and worsens fluid retention. Low urinary sodium reflects this impaired natriuresis and heightened neurohormonal activity, both associated with poor diuretic response and worse outcomes. Therefore, urinary sodium levels can be a useful marker for disease severity and therapeutic guidance in ADHF [8].

In a prior meta-analysis examining the association between urine sodium (urinary sodium concentration (UNa)) concentration, clinical measures of diuretic efficacy, and outcomes of ADHF, high UNa was associated with increased urinary output, greater weight loss, shorter duration of hospitalization, and lower odds of mortality [9]. However, these studies combined data from both spot UNa measurements and total sodium excretion. Spot UNa was collected at varying times across studies, ranging from one to two hours after diuretic administration to up to 72 hours after admission, potentially introducing confounding bias. We conducted a systematic review and meta-analysis to quantify mortality and clinical outcomes associated with spot UNa in patients with ADHF after the first void or within six hours of diuretic administration.

This article was previously presented as a meeting abstract at the 2025 ACC Annual Scientific Session & Expo (ACC.25) on March 31, 2025.

## Review

Methods

The systematic review and meta-analysis were performed and reported following the Cochrane Collaboration Handbook for Systematic Reviews of Interventions and the Preferred Reporting Items for Systematic Reviews and Meta-Analysis Statement guidelines. In addition, it was registered in the International Prospective Register of Systematic Reviews (CRD42024626912).

Data Source and Search Strategy

We systematically searched MEDLINE, Cochrane, and Embase databases from inception to May 2026. The search terms used included "acute heart failure", "acute decompensated heart failure", "natriuresis", and "spot urine sodium". Two authors (M.-D.I. and E.R.) independently performed the search and extracted the data following predefined search criteria. All search results were imported into Zotero (George Mason University, Fairfax, VA), where articles were screened and duplicates removed. Data regarding study design, patient characteristics, and outcomes were extracted into a structured Microsoft Excel spreadsheet (Microsoft Corporation, Redmond, WA). Any discrepancies were resolved through discussion until consensus was reached.

Eligibility Criteria

Studies were deemed eligible for inclusion if they met the following criteria: 1) randomized controlled trials or observational studies, 2) directly compared outcomes between patients with high and low UNa levels, 3) studied population included patients diagnosed with ADHF, and 4) spot UNa was collected either after the first void or within six hours of diuretic administration. Studies were excluded if they 1) did not specify the timing of spot UNa collection, 2) lacked any clinical endpoint of interest, and 3) were abstracts, case reports, or case series.

Endpoints

Our primary endpoints were mortality, worsening renal function, length of hospitalization/stay (LOS), and inotrope use.

Quality Assessment

The Cochrane tool for assessing Risk Of Bias In Nonrandomized Studies of Interventions (ROBINS-I) was utilized for the quality assessment [10]. Two authors (M.-D.I., E.R.) independently evaluated the risk of bias. Any discrepancies were addressed and resolved by consensus. The ROBINS-I assessment for all eight observational studies across seven domains has been presented: confounding, selection of participants, classification of interventions, deviations from intended interventions, missing data, outcome measurement, and selection of reported results. The overall risk of bias was rated as moderate for all eight observational studies.

Statistical Analysis

We used the Mantel-Haenszel random-effects model with odds ratios (ORs) and 95% confidence intervals (CIs) as the measure of effect size for binary endpoints. The restricted maximum-likelihood estimator was adopted to calculate heterogeneity variance τ² to account for considerable heterogeneity across studies. We assessed heterogeneity with Cochrane's Q statistic and Higgins and Thompson's I² statistic, with p ≤ 0.10 indicating statistical significance. We determined the consistency of the studies based on I² values of 0%, ≤ 25%, ≤ 50%, and > 50%, indicating no observed, low, moderate, and substantial heterogeneity, respectively. All tests were two-tailed, and a p value of <0.05 was considered statistically significant. All statistical analyses were performed using Review Manager 5.4.1 (The Cochrane Collaboration, London, UK; 2020).

Results

**Figure 1:**
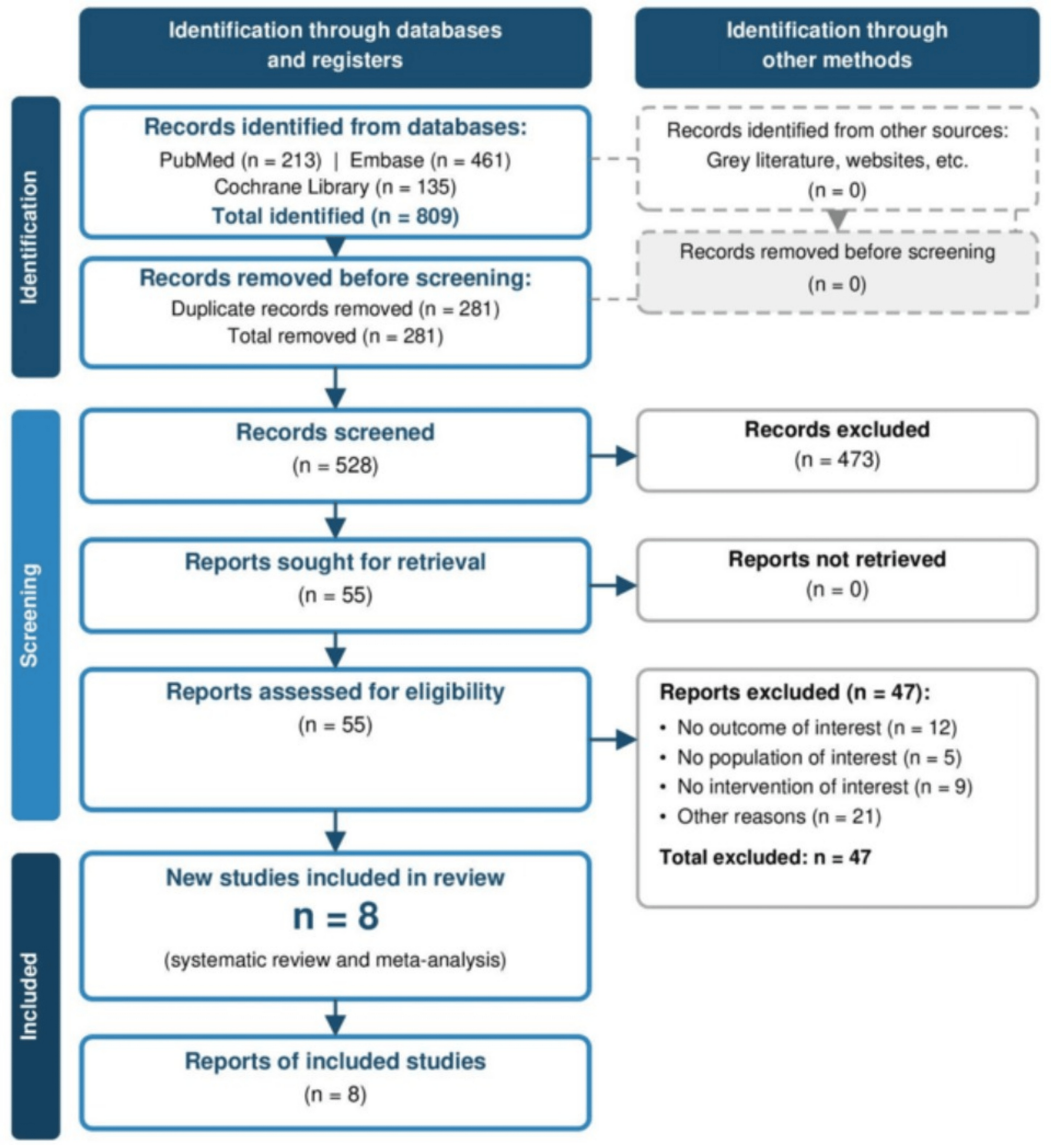
PRISMA flow diagram of study screening and selection PRISMA: Preferred Reporting Items for Systematic Reviews and Meta-Analysis

Our systematic search of MEDLINE, Cochrane, and Embase databases yielded 809 potential articles (Figure 1). After removing duplicates, 528 records were screened, of which 473 were excluded after title and abstract review. The remaining 55 full-text articles were assessed for eligibility; 47 were excluded for reasons including inappropriate UNa measurement timing, absence of a relevant clinical endpoint, or inappropriate study design (abstracts, case reports, case series). Eight studies satisfied all prespecified inclusion criteria and were retained for quantitative synthesis [11-18].

Patient Characteristics

We included 1,359 patients across eight studies, with 923 (67.9%) assigned to the high UNa group and 436 (32.1%) to the low UNa group. The mean age was 72 years, and 718 (52.8%) patients were men (Table 1). All eight studies were observational (prospective) in design. The definition of high vs. low UNa was heterogeneous across studies; the cutoff for high UNa ranged from 50 to 113 mEq/L. UNa was measured after the first postdiuretic void in two studies, at two hours postdiuretic in four studies, and at six hours postdiuretic in two studies. Mean patient age ranged from 67 to 75 years, left ventricular ejection fraction ranged from approximately 25% to 45%, and estimated glomerular filtration rate (eGFR) ranged from approximately 40 to 55 mL/minute/1.73 m². The variation in baseline characteristics and UNa cutoffs across studies reflects the real-world heterogeneity of ADHF populations and underscores the importance of interpreting effect estimates within their study-specific contexts.

**Table 1:** Baseline characteristics of included studies Values are represented as mean ± standard deviation unless otherwise stated. UNa cutoff denotes the threshold used to define high vs. low UNa in each study Obs: observational; UNa: urinary sodium; HTN: hypertension; DM2: type 2 diabetes mellitus; HiUNa: high urine sodium; LoUNa: low urine sodium; LVEF: left ventricular ejection fraction; eGFR: estimated glomerular filtration rate; N/A: not available

Study	Design	Spot UNa cutoff (mEq/L)	Patient (n)	Urine collection time (hours)	Male, n (%)	Age (years)	LVEF (%)	eGFR (mL/minute/1.73 m²)	HTN, n (%)	DM2, n (%)
		HiUNa/LoUNa	HiUNa/LoUNa	HiUNa/LoUNa	HiUNa/LoUNa	HiUNa/LoUNa	HiUNa/LoUNa	HiUNa/LoUNa	HiUNa/LoUNa	HiUNa/LoUNa
Cobo-Marcos et al. [11]	Obs (prospective)	50	82/20	6 hours	50/11	76 ± 10/76 ± 14	42 ± 19/48 ± 19	N/A	62/16	37/10
Luk et al. [12]	Obs (prospective)	60	72/31	First void	N/A	67 ± 12/68 ± 15	N/A	42 (29-59)/39 (29-54)	N/A	31 (43.1)/15 (48.4)
Biegus et al. [13]	Obs (prospective)	50	54/57	6 hours	45 (83)/42 (74)	65 ± 12/65 ± 14	N/A	N/A	44 (68.8)/12 (80)	21 (32.8)/10 (66.7)
Caravaca Pérez et al. [14]	Obs (prospective)	113	32/33	2 hours	19 (58)/21 (66)	65 ± 17/65 ± 14	41 ± 16/39 ± 16	69 ± 19/57 ± 20	24 (73)/23 (72)	12 (36)/8 (25)
García-Magallón et al. [15]	Obs (prospective)	70	52/21	2 hours	31 (59.6)/12 (57.1)	78 ± 11/71 ± 29	51 ± 15/48 ± 22	69.4 (44.1-86.2)/49.3 (28.0-63.2)	44 (84.6)/18 (85.7)	17 (32.7)/12 (57.1)
Honda et al. [16]	Obs (prospective)	74	444/225	First void	275 (62)/124 (55).	76.1 ± 11.7/75.5 ± 11.3	37.7 ± 16.6/38.3 ± 17.8	41.1 ± 17.7/35.6 ± 21.8	332 (75)/160 (71)	157 (35.3)/86 (38)
Londoño et al. [17]	Obs (prospective)	70	64/15	2 hours	N/A	67.7 (14.1)/74.2 (11.6)	31 (23-45)/30 (20-50)	N/A	44 (68.8)/12 (80)	21 (32.8)/10 (66.7)
Scatularo et al. [18]	Obs (prospective)	70	123/34	2 hours	70 (56.9)/18 (53)	74 ± 14/69 ± 15	44 ± 23/43 ± 21	N/A	98 (79.7)/23 (67)	44 (35.8)/13 (38)

Outcomes

**Figure 2:**
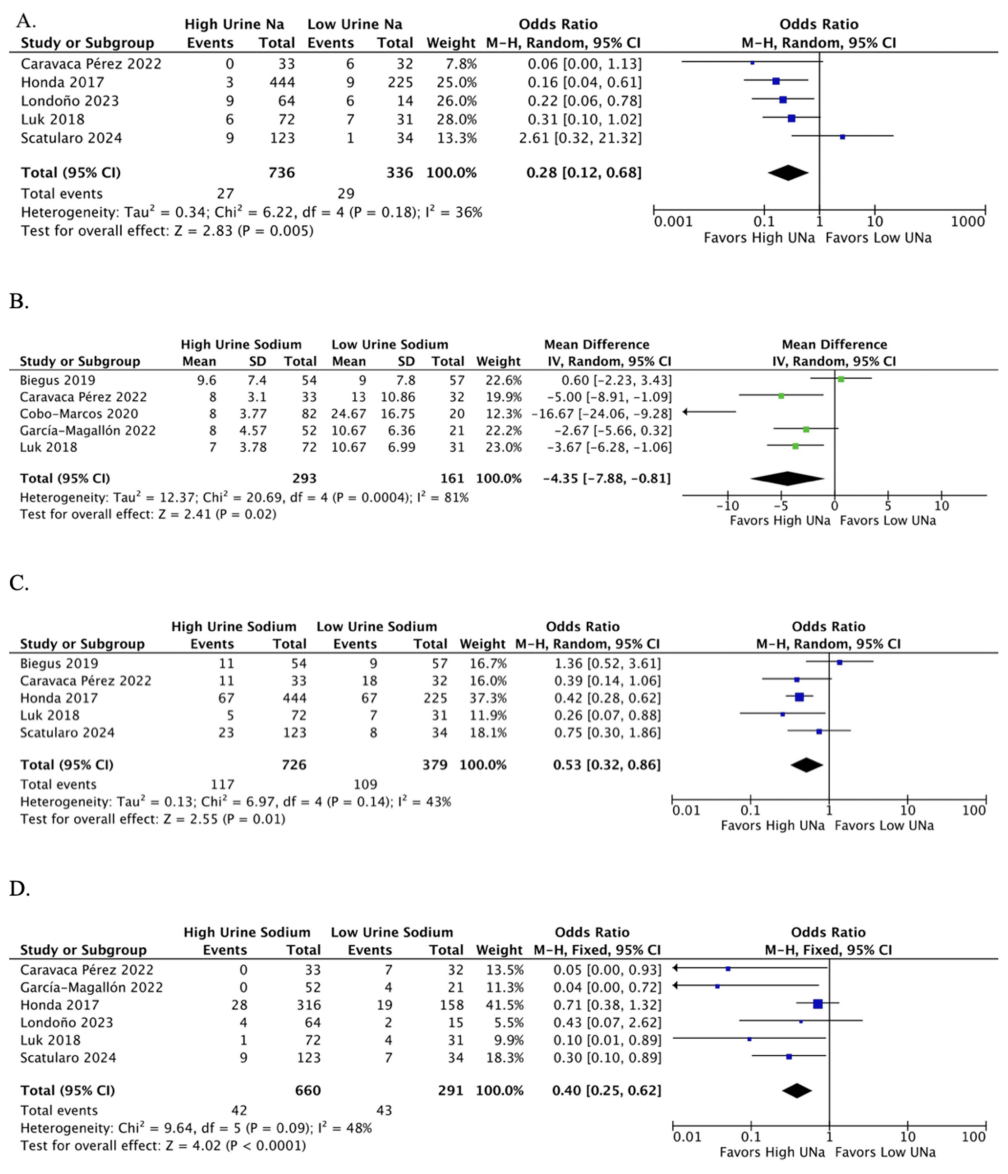
Primary endpoints Compared with low urinary sodium, high urinary sodium was associated with reduced mortality [12,14,16-18] (A), shorter length of hospitalization [11-15] (B), reduced worsening kidney function [12-14,16,18] (C), and reduced inotrope use [12,14-18] (D) in patients with acute decompensated heart failure. Forest plots generated using a random-effects model (Mantel-Haenszel method). Squares represent study-level odds ratios (or mean differences for B), with horizontal lines indicating 95% confidence intervals. Diamonds represent pooled estimates

In patients with ADHF, high UNa was associated with lower mortality (OR, 0.28; 95% CI, 0.12-0.68; Figure 2A), reduced inotrope use (OR, 0.40; 95% CI, 0.25-0.62; Figure 2D), decreased rates of worsening renal function (OR, 0.53; 95% CI, 0.32-0.86; Figure 2C), and shorter LOS (mean difference (MD), -4.35; 95% CI, -7.88 to -0.81; Figure 2B) compared with low UNa. Figure 2 presents forest plots for each of the four primary outcomes. Figure 2A displays the mortality analysis, which included five studies and demonstrated a statistically significant and clinically meaningful reduction in mortality with high UNa (OR, 0.28; 95% CI, 0.12-0.68; p = 0.005). Heterogeneity was moderate (I² not significant, τ² = 0.34). Figure 2B presents the LOS analysis, based on five studies, showing a mean reduction of 4.35 days (95% CI, -7.88 to -0.81; p = 0.02) in the high UNa group; heterogeneity was substantial (I² = 81%, p = 0.0004), reflecting variability in baseline LOS across study populations and healthcare systems. Figure 2C depicts the worsening renal function analysis from five studies, confirming a significant reduction in the odds of renal deterioration (OR, 0.53; 95% CI, 0.32-0.86; p = 0.01) with high UNa, with low-to-moderate heterogeneity (I² not significant, τ² = 0.13). Figure 2D shows the inotrope use analysis from six studies, demonstrating the strongest association: high UNa was associated with a 60% reduction in inotrope requirements (OR, 0.40; 95% CI, 0.25-0.62; p < 0.0001), with acceptable heterogeneity (I² = 48%, p = 0.09).

**Figure 3:**
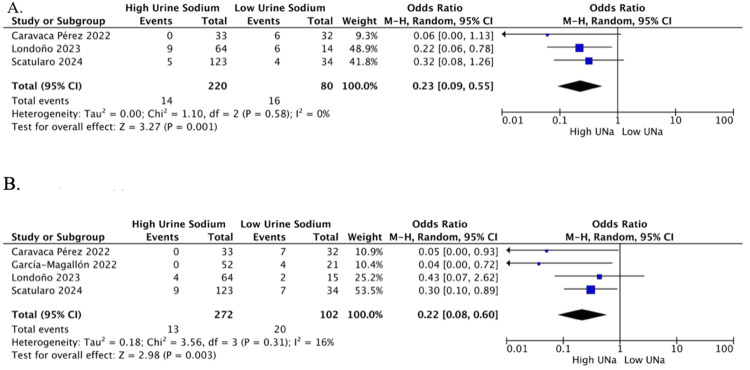
Subgroup analysis In the two-hour postdiuretic subgroup [14,15,17,18], high urinary sodium was associated with lower mortality [14,17,18] (A) and reduced inotrope use [14,15,17,18] (B) compared with low urinary sodium. Forest plots were generated using a random-effects model (Mantel-Haenszel method)

In a subgroup analysis restricted to studies measuring UNa at two hours after diuretic administration, high UNa was also associated with lower mortality (OR, 0.23; 95% CI, 0.09-0.55; Figure 3A), and reduced inotrope use (OR, 0.22; 95% CI, 0.08-0.60; Figure 3B). Figure 3 presents the two-hour subgroup forest plots. Figure 3A (mortality) is based on three studies and shows an even more pronounced mortality reduction at two hours (OR, 0.23; 95% CI, 0.09-0.55; p = 0.001), with no significant heterogeneity (τ² = 0.00). Figure 3B (inotrope use) is based on four studies and demonstrates the most pronounced effect in the subgroup analysis (OR, 0.22; 95% CI, 0.08-0.60; p = 0.003), with moderate heterogeneity. The consistency of findings across both the overall and two-hour subgroup analyses supports the robustness of early spot UNa as a prognostic biomarker and strengthens the pharmacodynamic rationale for a two-hour assessment window.

Quality Assessment

Table 2 presents the risk-of-bias assessments for the included studies.

**Table 2:** Risk of bias - observational studies (ROBINS-I, n = 8) Domains: 1: confounding - moderate in all studies, reflecting incomplete control of dietary sodium intake and concomitant medications; 2-7: rated as low for most studies. Overall risk of bias was moderate in all eight observational studies Low: low risk of bias; moderate: moderate risk of bias; high: high risk of bias; ROBINS-I: Risk of Bias In Nonrandomized Studies of Interventions

Study	Confounding	Selection of participants	Classification of interventions	Deviations from intended interventions	Missing data	Outcome measurement	Selection of reported results	Overall
Cobo-Marcos et al. [11]	Moderate	Low	Low	Low	Low	Low	Low	Moderate
Luk et al. [12]	Moderate	Low	Low	Low	Low	Low	Low	Moderate
Biegus et al. [13]	Moderate	Low	Low	Low	Low	Low	Moderate	Moderate
Caravaca Pérez et al. [14]	Moderate	Low	Low	Low	Moderate	Low	Moderate	Moderate
García-Magallón et al. [15]	Moderate	Low	Low	Low	Low	Low	Low	Moderate
Honda et al. [16]	Moderate	Low	Low	Low	Low	Low	Low	Moderate
Londoño et al. [17]	Moderate	Moderate	Low	Low	Moderate	Low	Moderate	Moderate
Scatularo et al. [18]	Moderate	Low	Low	Low	Low	Low	Low	Moderate

Discussion

This comprehensive systematic review and meta-analysis of 1,359 patients examined the prognostic value of spot UNa in the management of ADHF. We found that higher UNa after the first void or within the first six hours of diuretic therapy initiation was associated with lower odds of mortality, shorter LOS, lower inotrope use, and lower rates of renal dysfunction compared with low UNa. To the best of our knowledge, this meta-analysis is the first to analyze the prognostic value of spot UNa using data exclusively from studies where spot UNa was measured after the first void or within six hours of diuretic therapy in AHF patients.

A recent position statement by the Heart Failure Association of the European Society of Cardiology (ESC) recommends doubling the intravenous diuretic dose in patients with UNa levels below 50-70 mmol/L two hours after treatment initiation or with an hourly urine output of less than 100-150 mL during the first six hours of treatment [1]. Previous meta-analyses have demonstrated that higher UNa levels are associated with better outcomes [9]. However, those analyses combined data from both spot UNa measurements and total sodium excretion, with spot UNa collected across a wide temporal window, from one to two hours after diuretic administration to up to 72 hours after admission. In our meta-analysis, we focused exclusively on studies measuring spot UNa from urine collected after the first void or within six hours of diuretic administration. This threshold aligns with the pharmacodynamic properties of loop diuretics, which have a half-life of approximately 1.5-2 hours and complete their natriuretic effect within six hours [8]. Our review supports assessing UNa excretion from a spot urine sample, an easier, quicker, and less resource-intensive approach than 24-hour urine collection, providing a rapid and clinically actionable measure of diuretic response within six hours of initiating therapy.

The association between high UNa and a 72% reduction in the odds of mortality (OR, 0.28; 95% CI, 0.12-0.68) represents the most clinically consequential finding of this analysis. This magnitude of association was consistent across the two-hour subgroup analysis (OR, 0.23; 95% CI, 0.09-0.55), lending biological plausibility to the finding. In ADHF, low UNa reflects impaired natriuresis driven by heightened neurohormonal activation, principally elevated aldosterone and angiotensin II, which promote avid tubular sodium reabsorption and attenuate the response to loop diuretics. High UNa, conversely, signals adequate distal tubular drug delivery, sufficient effective arterial filling pressure, and a more favorable hemodynamic profile, conditions that collectively portend a less severe in-hospital course and lower short-term mortality.

Effective decongestion and prevention of adverse outcomes are the primary goals in managing ADHF [1,19]. Current guidelines recommend parenteral loop diuretics for symptom relief in ADHF, with initial doses adjusted based on prior oral diuretic intake [1]. However, despite the recognized efficacy of loop diuretics, approximately one-third of patients discharged after ADHF episodes exhibit residual signs or symptoms of congestion, leading to rehospitalization and death [19]. Efforts have been made to optimize diuretic dosing strategies to achieve full decongestion by discharge. Various parameters, including symptoms, vital signs, diuresis, weight changes, serum electrolytes, renal function, CA125 levels on admission, and lung ultrasound-guided treatment, have been proposed or utilized [7,20]. Nevertheless, many of these measures lack the accuracy to identify ineffective diuretic responses. In contrast, urinary sodium is a direct and quantifiable marker of natriuretic response that captures both renal tubular sodium handling and the effectiveness of diuretic therapy, offering an objective and reproducible assessment of diuresis. This reduces reliance on surrogate or observer-dependent measures and provides a standardized approach for evaluating decongestion in acute heart failure.

Lowering hospital readmission rates is a major priority for healthcare systems worldwide [21,22]. Previous studies have shown that longer LOS for ADHF is associated with higher 30-day all-cause readmission risk and mortality [22-24]. Our review demonstrates that a higher UNa is associated with a significant decrease in LOS (MD, -4.35 days; 95% CI, -7.88 to -0.81), underscoring the potential of UNa-guided diuretic titration to reduce the period of hospitalization. This could allow early identification of poor diuretic responders, prompt therapeutic escalation, and reduce the proportion of patients discharged with residual congestion. Earlier and more effective decongestion, reflected by an adequate early natriuretic response, has the potential to lower hospital readmission rates and reduce the substantial economic burden associated with ADHF hospitalizations.

Worsening kidney function during ADHF hospitalizations is common due to multiple cardiorenal mechanisms, including venous congestion, reduced forward flow, and neurohormonal activation [25,26]. To avoid this complication of aggressive diuresis, many ADHF patients are discharged with residual congestion, which in turn leads to early rehospitalization [14]. In this systematic review and meta-analysis, high UNa was associated with a significant reduction in the rates of worsening renal function (OR, 0.53; 95% CI, 0.32-0.86), challenging the longstanding clinical assumption that aggressive diuresis is uniformly injurious to renal function in ADHF. This finding is consistent with evidence that venous congestion, rather than reduced forward flow per se, is the dominant driver of acute cardiorenal injury in most patients with decompensated heart failure. Effective early decongestion, as reflected by an adequate natriuretic response, may therefore be renoprotective rather than nephrotoxic. The Heart Failure Association of the ESC has endorsed the use of spot UNa measurement to assess diuretic effectiveness more accurately than relying solely on serum creatinine and eGFR [27].

Inotropes are required in patients with ADHF complicated by systolic dysfunction and hypoperfusion; however, their use is associated with an increased risk of arrhythmias and higher mortality [28]. In this meta-analysis, higher urinary sodium levels were associated with a 60% reduction in the need for inotropic therapy (OR, 0.40; 95% CI, 0.25-0.62), a finding that was even more pronounced in the two-hour subgroup analysis (OR, 0.22; 95% CI, 0.08-0.60). This suggests that an adequate natriuretic response may reflect sufficient hemodynamic reserve to achieve stability through decongestion alone, potentially averting escalation to high-risk inotropic agents in borderline cases. In clinical practice, this finding supports a therapeutic sequence in which aggressive, protocol-driven diuresis, monitored by early UNa measurement, is trialed before inotropes are initiated in patients without overt cardiogenic shock.

Natriuretic peptides (B-type natriuretic peptide, N-terminal pro-B-type natriuretic peptide) remain the cornerstone of risk stratification in ADHF, but their utility for tracking acute decongestion is limited by delayed kinetics, levels may not fall appreciably for 24-72 hours after effective diuresis, and by confounding from renal dysfunction, obesity, and atrial fibrillation [7]. Serum creatinine and eGFR do not distinguish appropriate hemodynamic adaptation from diuretic-induced nephrotoxicity. Spot UNa, by contrast, is quantitative, rapidly available, inexpensive, and mechanistically direct, measuring the immediate pharmacological interaction between the loop diuretic and its tubular target. This positions spot UNa as a complementary, rather than competing, tool within the ADHF biomarker armamentarium. An integrated model combining early UNa with natriuretic peptide trajectories, clinical congestion scores, and renal function markers may ultimately provide more precise and actionable risk stratification than any single biomarker in isolation.

Strengths and Limitations

This meta-analysis synthesizes evidence from multiple studies, providing a more robust assessment of the relationship between urinary sodium and outcomes in ADHF. The findings are clinically relevant, highlighting UNa as a simple, widely available biomarker that may support risk stratification and therapeutic guidance in routine practice. The consistent association between higher UNa and favorable outcomes, including reduced mortality, inotrope use, and shorter hospitalization, across both the full analysis and the prespecified two-hour subgroup reinforces the biological plausibility of natriuretic response as a prognostic marker. Moreover, by providing pooled effect estimates, the analysis enhances the precision of outcome assessment and underscores potential treatment implications, particularly regarding diuretic optimization and reduction of reliance on high-risk interventions. Critically, the restriction to studies measuring UNa within six hours of diuretic administration represents a methodological advance over prior meta-analyses, improving comparability across studies and alignment with the pharmacodynamic window of loop diuretics.

This study has several limitations. Most of the included studies were observational and single-center in design, raising the potential for selection bias and residual confounding that cannot be fully addressed through pooled analysis. There was marked variability in the cutoff values used to define low and high urinary sodium (ranging from 50 to 113 mEq/L), and spot urine collection methods were not standardized across studies. In addition, factors known to influence UNa levels, such as dietary salt intake and concomitant medications including corticosteroids, antibiotics, and antidepressants, were not consistently accounted for. It was also unclear whether patients were instructed to empty their bladder prior to diuretic administration, which could have led to mixing of pre- and postdiuretic urine and confounded sodium measurements. Furthermore, the growing use of sodium-glucose cotransporter-2 inhibitors in heart failure, agents that independently exert a natriuretic effect, was not addressed in the included studies, limiting generalizability to contemporary practice. The predominantly European and East Asian study populations and the relative underrepresentation of patients with heart failure with preserved ejection fraction also temper the generalizability of findings.

## Conclusions

In this systematic review and meta-analysis of 1,359 patients with ADHF, spot urinary sodium measured after the first void or within six hours of loop diuretic administration was independently associated with lower odds of in-hospital mortality (OR, 0.28; 95% CI, 0.12-0.68), reduced inotrope requirement (OR, 0.40; 95% CI, 0.25-0.62), decreased rates of worsening renal function (OR, 0.53; 95% CI, 0.32-0.86), and shorter LOS (MD, -4.35 days; 95% CI, -7.88 to -0.81) compared with low urinary sodium. These findings were consistent in the prespecified two-hour subgroup analysis, supporting the pharmacodynamic rationale for early natriuretic response assessment and strengthening the evidence base for current Heart Failure Association of the ESC guidance on spot urine sodium-guided diuretic titration. Spot urine sodium is a practical, inexpensive, and mechanistically direct biomarker that can be readily integrated into routine ADHF management to identify diuretic nonresponders and guide timely therapeutic escalation. Large-scale, multicenter randomized trials with standardized collection protocols are warranted to confirm these findings and establish an optimal UNa threshold for clinical decision-making.
